# Symbolic Partial-Order Execution for Testing Multi-Threaded Programs

**DOI:** 10.1007/978-3-030-53288-8_18

**Published:** 2020-06-13

**Authors:** Daniel Schemmel, Julian Büning, César Rodríguez, David Laprell, Klaus Wehrle

**Affiliations:** 8 Microsoft Research Lab, Redmond, WA USA; 9 University of Southern California, Los Angeles, CA USA; 10 RWTH Aachen University, Aachen, Germany; 11 Diffblue Ltd., Oxford, UK; 12 Université Paris 13, Sorbonne Paris Cité, CNRS, Paris, France

**Keywords:** Software testing, Symbolic Execution, Partial-Order Reduction

## Abstract

We describe a technique for systematic testing of multi-threaded programs. We combine Quasi-Optimal Partial-Order Reduction, a state-of-the-art technique that tackles path explosion due to interleaving non-determinism, with symbolic execution to handle data non-determinism. Our technique iteratively and exhaustively finds all executions of the program. It represents program executions using partial orders and finds the next execution using an underlying unfolding semantics. We avoid the exploration of redundant program traces using cutoff events. We implemented our technique as an extension of KLEE and evaluated it on a set of large multi-threaded C programs. Our experiments found several previously undiscovered bugs and undefined behaviors in memcached and GNU sort, showing that the new method is capable of finding bugs in industrial-size benchmarks.

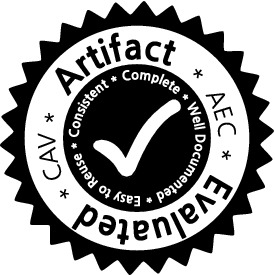



## Introduction

Advances in formal testing and the increased availability of affordable concurrency have spawned two opposing trends: While it has become possible to analyze increasingly complex sequential programs in new and powerful ways, many projects are now embracing parallel processing to fully exploit modern hardware, thus raising the bar for practically useful formal testing. In order to make formal testing accessible to software developers working on parallel programs, two main problems need to be solved. Firstly, a significant portion of the API in concurrency libraries such as libpthread must be supported. Secondly, the analysis must be accessible to non-experts in formal verification. Currently, this niche is mostly occupied by manual and fuzz testing, oftentimes combined with dynamic concurrency checkers such as ThreadSanitizer [45] or Helgrind [2].

Data non-determinism in sequential and concurrent programs, and scheduling non-determinism are two major sources of path explosion in program analysis. *Symbolic execution* [10, 11, 22, 29, 38] is a technique to reason about input data in sequential programs. It is capable of dealing with real-world programs. Partial-Order Reductions (PORs) [5, 19, 20, 41] are a large family of techniques to explore a reduced number of thread interleavings without missing any relevant behavior.

In this paper we propose a technique that combines symbolic execution and a Quasi-Optimal POR [35]. In essence, our approach (1) runs the program using a symbolic executor, (2) builds a partial order representing the occurrence of POSIX threading synchronization primitives (library functions pthread_*) seen during that execution, (3) adds the partial order to an underlying tree-like, unfolding [32, 41] data structure, (4) computes the first events of the next partial orders to explore, and (5) selects a new partial order to explore and starts again. We use cutoff events [32] to prune the exploration of different traces that reach the same state, thus natively dealing with non-terminating executions.

We implemented our technique as an extension of KLEE. During the evaluation of this prototype we found nine bugs (that we attribute to four root causes) in the production version of memcached. All of these bugs have since been confirmed by the memcached maintainers and are fixed as of version 1.5.21. Our tool handles a significant portion of the POSIX threading API [4], including barriers, mutexes and condition variables without being significantly harder to use than common fuzz testing tools.

The main challenge that our approach needs to address is that of scalability in the face of an enormous state space. We tackle this challenge by detecting whenever any two Mazurkiewicz traces reach the same program state to only further explore one of them. Additionally, we exploit the fact that data races on non-atomic variables cause undefined behavior in C [25, § 5.1.2.4/35], which means that any unsynchronized memory access is, strictly speaking, a bug. By adding a data race detection algorithm, we can thereby restrict thread scheduling decisions to synchronization primitives, such as operations on mutexes and condition variables, which significantly reduces the state space.

This work has three core contributions, the combination of which enables the analysis of real-world multi-threaded programs (see also Sect. 6 for related work):

1. A partial-order reduction algorithm capable of handling real-world POSIX programs that use an arbitrary amount of threads, mutexes and condition variables. Our algorithm continues analysis in the face of deadlocks.
2. A cutoff algorithm that recognizes whenever two Mazurkiewicz traces reach the same program state, as identified by its actual memory contents. This significantly prunes the search space and even enables the partial-order reduction to deal with non-terminating executions.
3. An implementation that finds real-world bugs.

We also present an extended, more in-depth version of this paper [42].

## Overview

The technique proposed in this paper can be described as a process of 5 conceptual steps, each of which we describe in a section below:

**Fig. 1.**
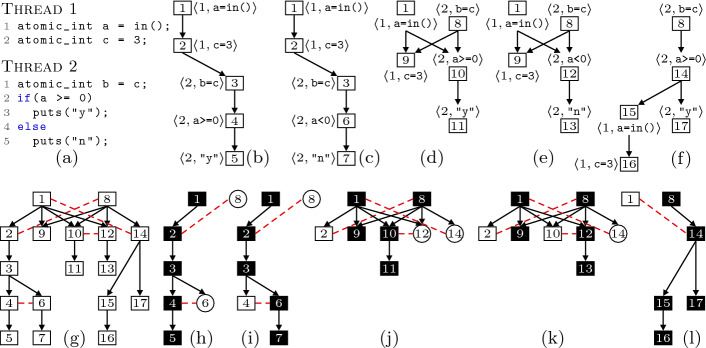
A program (a) with its 5 partial-order runs (b–f), its unfolding (g) and the 5 steps used by our algorithm to visit the unfolding (h–l).

### Sequential Executions

Consider the program shown in Fig. 1a. Assume that all variables are initially set to zero. The statement a = in() initializes variable a non-deterministically. A *run* of the program is a sequence of *actions*, i.e., pairs $\langle i,s\rangle$ where $i \in \mathbb {N}$ identifies a thread that executes a statement $s$. For instance, the sequence

$$ \sigma _1 \mathrel {:=}\langle 1, {\texttt {a=in()}}\rangle , \langle 1, {\texttt {c=3}}\rangle , \langle 2, {\texttt {b=c}}\rangle , \langle 2, {\texttt {a<0}}\rangle , \langle 2, {\texttt {puts("n")}}\rangle $$

is a run of Fig. 1a. This run represents all program paths where both statements of thread 1 run before the statements of thread 2, and where the statement a = in() initializes variable a to a negative number. In our notion of run, concurrency is represented explicitly (via thread identifiers) and data non-determinism is represented symbolically (via constraints on program variables). To keep things simple the example only has atomic integers (implicitly guarded by locks) instead of POSIX synchronization primitives.

### Independence Between Actions and Partial-Order Runs

Many POR techniques use a notion called *independence* [20] to avoid exploring concurrent interleavings that lead to the same state. An independence relation associates pairs of actions that commute (running them in either order results in the same state). For illustration purposes, in Fig. 1 let us consider two actions as *dependent* iff either both of them belong to the same thread or one of them writes into a variable which is read/written by the other. Furthermore, two actions will be *independent* iff they are not dependent.

A sequential run of the program can be viewed as a partial order when we take into account the independence of actions. These partial orders are known as *dependency graphs* in Mazurkiewicz trace theory [31] and as *partial-order runs* in this paper. Figures 1b to 1f show all the partial-order runs of Fig. 1a. The partial-order run associated to the run $\sigma _1$ above is Fig. 1c. For

$$ \sigma _2 \mathrel {:=}\langle 2, {\texttt {b=c}}\rangle , \langle 2, {\texttt {a>=0}}\rangle , \langle 1, {\texttt {a=in()}}\rangle , \langle 2, {\texttt {puts("y")}}\rangle , \langle 1, {\texttt {c=3}}\rangle , $$

we get the partial order shown in Fig. 1f.

### Unfolding: Merging the Partial Orders

An unfolding [16, 32, 37] is a tree-like structure that uses partial orders to represent concurrent executions and conflict relations to represent thread interference and data non-determinism. We can define unfolding semantics for programs in two conceptual steps: (1) identify isomorphic events that occur in different partial-order runs; (2) bind the partial orders together using a conflict relation.

Two events are *isomorphic* when they are structurally equivalent, i.e., they have the same label (run the same action) and their causal (i.e., happens-before) predecessors are (transitively) isomorphic. The number within every event in Figs. 1b to 1f identifies isomorphic events.

Isomorphic events from different partial orders can be merged together using a conflict relation for the un-merged parts of those partial orders. To understand why conflict is necessary, consider the set of events $C \mathrel {:=}{\{ 1, 2 \mathclose \}}$. It obviously represents part of a partial-order run (Fig. 1c, for instance). Similarly, events $C' \mathrel {:=}{\{ 1, 8, 9 \mathclose \}}$ represent (part of) a run. However, their union $C \cup C'$ does not represent any run, because (1) it does not describe what happens-before relation exists between the dependent actions of events 2 and 8, and (2) it executes the statement c=3 twice. Unfoldings fix this problem by introducing a *conflict* relation between events. Conflicts are to unfoldings what branches are to trees. If we declare that events 2 and 8 are in conflict, then any conflict-free (and causally-closed) subset of $C \cup C'$ is exactly one of the original partial orders. This lets us merge the common parts of multiple partial orders without losing track of the original partial orders.

Figure 1g represents the unfolding of the program (after merging all 5 partial-order runs). Conflicts between events are represented by dashed red lines. Each original partial order can be retrieved by taking a ($\subseteq$-maximal) set of events which is conflict-free (no two events in conflict are in the set) and causally closed (if you take some event, then also take all its causal predecessors).

For instance, the partial order in Fig. 1d can be retrieved by resolving the conflicts between events 1 vs. 14, 2 vs. 8, 10 vs. 12 in favor of, resp., 1, 8, 10. Resolving in favor of 1 means that events 14 to 17 cannot be selected, because they causally succeed 14. Similarly, resolving in favor of 8 and 10 means that only events 9 and 11 remain eligible, which hold no conflicts among them—all other events are causal successors of either 2 or 12.

### Exploring the Unfolding

Since the unfolding represents all runs of the program via a set of compactly-merged, prefix-sharing partial orders, enumerating all the behaviors of the program reduces to exploring all partial-order runs represented in its unfolding. Our algorithm iteratively enumerates all $\subseteq$-maximal partial-order runs.

In simplified terms, it proceeds as follows. Initially we explore the black events shown in Fig. 1h, therefore exploring the run shown in Fig. 1b. We discover the next partial order by computing the so-called *conflicting extensions* of the current partial order. These are, intuitively, events in conflict with some event in our current partial order but such that all its causal predecessors are in our current partial order. In Fig. 1h these are shown in circles, events 8 and 6.

We now find the next partial order by (1) selecting a conflicting extension, say event 6, (2) removing all events in conflict with the selected extension and their causal successors, in this case events 4 and 5, and (3) expanding the partial order until it becomes maximal, thus exploring the partial order Fig. 1c, shown as the black events of Fig. 1i. Next we select event 8 (removing 2 and its causal successors) and explore the partial order Fig. 1d, shown as the black events of Fig. 1j. Note that this reveals two new conflicting extensions that were hidden until now, events 12 and 14 (hidden because 8 is a causal predecessor of them, but was not in our partial order). Selecting either of the two extensions makes the algorithm explore the last two partial orders.

### Cutoff Events: Pruning the Unfolding

When the program has non-terminating runs, its unfolding will contain infinite partial orders and the algorithm above will not finish. To analyze non-terminating programs we use *cutoff events* [32]. In short, certain events do not need to be explored because they reach the same state as another event that has been already explored using a shorter (partial-order) run. Our algorithm prunes the unfolding at these cutoff events, thus handling terminating and non-terminating programs that repeatedly reach the same state.

## Main Algorithm

This section formally describes the approach presented in this paper.

### Programs, Actions, and Runs

Let $P \mathrel {:=}\langle T, \mathcal {L}, \mathcal {C}\rangle$ represent a (possibly non-terminating) multi-threaded POSIX C program, where $T$ is the set of statements, $\mathcal {L}$ is the set of POSIX mutexes used in the program, and $\mathcal {C}$ is the set of condition variables. This is a deliberately simplified presentation of our program syntax, see [42] for full details. We represent the behavior of each statement in *P* by an *action*, i.e., a pair $\langle i, b\rangle$ in $A \subseteq \mathbb {N}\times B$, where $i \ge 1$ identifies the thread executing the statement and *b* is the *effect* of the statement. We consider the following effects:

$$\begin{aligned} B \mathrel {:=}&~({\{ \textsf {loc} \mathclose \}} \times T) \cup ({\{ \textsf {acq},\textsf {rel} \mathclose \}} \times \mathcal {L}) \cup ({\{ \textsf {sig} \mathclose \}} \times \mathcal {C}\times \mathbb {N}) \\ \cup&~({\{ \textsf {bro} \mathclose \}} \times \mathcal {C}\times 2^\mathbb {N}) \cup ({\{ \textsf {w}_1,\textsf {w}_2 \mathclose \}} \times \mathcal {C}\times \mathcal {L}) \end{aligned}$$

Below we informally explain the intent of an effect and how actions of different effects interleave with each other. In [42] we use *actions* and *effects* to define labeled transition system semantics to *P*. Below we also (informally) define an independence relation (see Sect. 2.2) between actions.

**Fig. 2.**
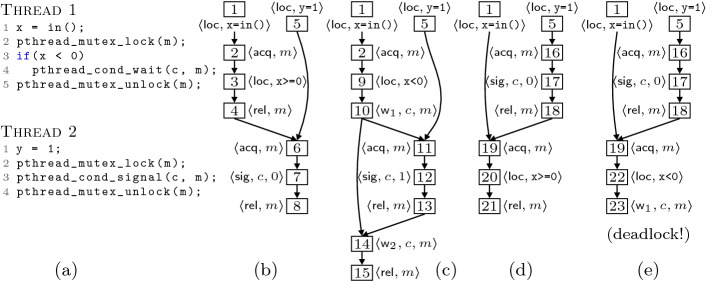
A program and its four partial-order runs.

*Local Actions.* An action $\langle i, \langle \textsf {loc}, t\rangle \rangle$ represents the execution of a *local* statement $t$ from thread $i$, i.e., a statement which manipulates local variables. For instance, the actions labeling events 1 and 3 in Fig. 2b are local actions. Note that local actions do not interfere with actions of other threads. Consequently, they are only dependent on actions of the same thread.

*Mutex Lock/Unlock.* Actions $\langle i, \langle \textsf {acq}, l\rangle \rangle$ and $\langle i, \langle \textsf {rel}, l\rangle \rangle$ respectively represent that thread $i$ locks or unlocks mutex $l \in \mathcal {L}$. The semantics of these actions correspond to the so-called NORMAL mutexes in the POSIX standard [4]. Actions of $\langle \textsf {acq}, l\rangle$ or $\langle \textsf {rel}, l\rangle$ effect are only dependent on actions whose effect is an operation on the same mutex $l$ ($\textsf {acq}$, $\textsf {rel}$, $\textsf {w}_1$ or $\textsf {w}_2$, see below). For instance the action of event 4 ($\textsf {rel}$) in Fig. 2b depends on the action of event 6 ($\textsf {acq}$).

*Wait on Condition Variables.* The occurrence of a pthread_cond_wait(c, l) statement is represented by two separate actions of effect  and . An action  represents that thread *i* has atomically released the lock *l* *and* started waiting on condition variable *c*. An action  indicates that thread *i* has been woken up by a *signal* or *broadcast* operation on *c* *and* that it successfully re-acquired mutex *l*. For instance the action $\langle 1, \langle \textsf {w}_1, c, m\rangle \rangle$ of event 10 in Fig. 2c represents that thread 1 has released mutex *m* and is waiting for *c* to be signaled. After the signal happens (event 12) the action $\langle 1, \langle \textsf {w}_2, c, m\rangle \rangle$ of event 14 represents that thread 1 wakes up and re-acquires mutex *m*. An action $\langle i, \langle \textsf {w}_1, c, l\rangle \rangle$ is dependent on any action whose effect operates on mutex *l* ($\textsf {acq}$, $\textsf {rel}$, $\textsf {w}_1$ or $\textsf {w}_2$) as well as signals directed to thread *i* ($\langle \textsf {sig}, c, i\rangle$, see below), lost signals ($\langle \textsf {sig}, c, 0\rangle$, see below), and any broadcast ($\langle \textsf {bro}, c, W\rangle$ for any $W \subseteq \mathbb {N}$, see below). Similarly, an action $\langle i, \langle \textsf {w}_2, c,l\rangle \rangle$ is dependent on any action whose effect operates on lock *l* as well as signals and broadcasts directed to thread *i* (that is, either $\langle \textsf {sig}, c, i\rangle$ or $\langle \textsf {bro}, c, W\rangle$ when $i \in W$).

*Signal/Broadcast on Condition Variables.* An action $\langle i, \langle \textsf {sig}, c, j\rangle \rangle$, with $j \ge 0$ indicates that thread *i* executed a pthread_cond_signal(c) statement. If $j = 0$ then no thread was waiting on condition variable *c*, and the *signal* had no effect, as per the POSIX semantics. We refer to these as *lost signals*. Example: events 7 and 17 in Fig. 2b and 2d are labeled by lost signals. In both cases thread 1 was not waiting on the condition variable when the signal happened. However, when $j \ge 1$ the action represents that thread *j* wakes up by this signal. Whenever a signal wakes up a thread $j \ge 1$, we can always find a (unique) $\textsf {w}_1$ action of thread *j* that happened before the signal and a unique $\textsf {w}_2$ action in thread *j* that happens after the signal. For instance, event 12 in Fig. 2c signals thread 1, which went sleeping in the $\textsf {w}_1$ event 10 and wakes up in the $\textsf {w}_2$ event 14. Similarly, an action $\langle i, \langle \textsf {bro}, c, W\rangle \rangle$, with $W \subseteq \mathbb {N}$ indicates that thread *i* executed a pthread_cond_broadcast(c) statement and any thread *j* such that $j \in W$ was woken up. If $W = \emptyset$, then no thread was waiting on condition variable *c* (*lost broadcast*). Lost signals and broadcasts on *c* depend on any action of $\langle \textsf {w}_1, c, \cdot \rangle$ effect as well as any non-lost signal/broadcast on *c*. Non-lost signals and broadcasts on *c* that wake up thread *j* depend^1^ on $\textsf {w}_1$ and $\textsf {w}_2$ actions of thread *j* as well as any signal/broadcast (lost or not) on the same condition variable.

A *run* of *P* is a sequence of actions in $A^*$ which respects the constraints stated above for actions. For instance, a run for the program shown in Fig. 2a is the sequence of actions which labels *any* topological order of the events shown in any partial order in Fig. 2b to 2e. The sequence below,is a run of Fig. 2a. Naturally, if $\sigma \in A^*$ is a run, any prefix of $\sigma$ is also a run. Runs explicitly represent concurrency, using thread identifiers, and symbolically represent data non-determinism, using constraints, as illustrated by the 1st and 4th actions of the run above. We let $\mathop { runs } (P)$ denote the set of all runs of *P*.

A *concrete state of* *P* is a tuple that represents, intuitively, the program counters of each thread, the values of all memory locations, the mutexes locked by each thread, and, for each condition variable, the set of threads waiting for it (see [42] for a formal definition). Since runs represent operations on symbolic data, they reach a symbolic state, which conceptually corresponds to a set of concrete states of *P*.

The *state of a run* $\sigma$, written $\mathop { state } (\sigma )$, is the set of all concrete states of *P* that are reachable when the program executes the run $\sigma$. For instance, the run $\sigma '$ given above reaches a state consisting on all program states where y is 1, x is a non-negative number, thread 2 owns mutex m and its instruction pointer is at line 3, and thread 1 has finished. We let $\mathop { reach } (P) \mathrel {:=}\bigcup _{\sigma \in \mathop { runs } (P)} \mathop { state } (\sigma )$ denote the set of all *reachable states* of *P*.

### Independence

In the previous section, given an action $a \in A$ we informally defined the set of actions which are *dependent* on *a*, therefore indirectly defining an *independence relation*. We now show that this relation is a *valid independence* [19, 41]. Intuitively, an independence relation is *valid* when every pair of actions it declares as independent can be executed in any order while still producing the same state.

Our independence relation is valid only for *data-race-free* programs. We say that *P* is *data-race-free* iff any two local actions $a \mathrel {:=}\langle i, \langle \textsf {loc}, t\rangle \rangle$ and $a' \mathrel {:=}\langle i', \langle \textsf {loc}, t'\rangle \rangle$ from different threads ($i \ne i'$) commute at every reachable state of *P*. See [42] for additional details. This ensures that local statements of different threads of *P* modify the memory without interfering each other.

#### Theorem 1

If *P* is data-race-free, then the independence relation defined in Sect. 3.1 is valid.

#### Proof

See [42].

Our technique does not use data races as a source of thread interference for partial-order reduction. It will not explore two execution orders for the two statements that exhibit a data race. However, it can be used to detect and report data races found during the POR exploration, as we will see in Sect. 4.4.

### Partial-Order Runs

A *labeled partial-order* (LPO) is a tuple $\langle X, {<}, h\rangle$ where $X$ is a set of *events*, ${<} \subseteq X \times X$ is a *causality* (a.k.a., *happens-before*) relation, and $h :X \rightarrow A$ labels each event by an *action* in $A$.

A *partial-order run* of *P* is an LPO that represents a run of *P* without enforcing an order of execution on actions that are independent. All partial-order runs of Fig. 2a are shown in Fig. 2b to 2e.

Given a run $\sigma$ of *P*, we obtain the corresponding partial-order run $\mathcal {E}_\sigma \mathrel {:=}\langle E, {<}, h\rangle$ by the following procedure: (1) initialize $\mathcal {E}_\sigma$ to be the only totally-ordered LPO that consists of $|\sigma |$ events where the i-th event is labeled by the i-th action of $\sigma$; (2) for every two events $e, e'$ such that $e < e'$, remove the pair $\langle e,e'\rangle$ from < if *h*(*e*) is independent from $h(e')$; (3) restore transitivity in < (i.e., if $e < e'$ and $e' < e''$, then add $\langle e, e''\rangle$ to <). The resulting LPO is a partial-order run of *P*.

Furthermore, the originating run $\sigma$ is an *interleaving* of $\mathcal {E}_\sigma$. Given some LPO $\mathcal {E}\mathrel {:=}\langle E, {<}, h\rangle$, an interleaving of $\mathcal {E}$ is the sequence that labels any topological ordering of $\mathcal {E}$. Formally, it is any sequence $h(e_1), \ldots , h(e_n)$ such that $E = {\{ e_1, \ldots , e_n \mathclose \}}$ and $e_i< e_j \implies i < j$. We let $\mathop { inter } (\mathcal {E})$ denote the set of all interleavings of $\mathcal {E}$. Given a partial-order run $\mathcal {E}$ of *P*, the interleavings $\mathop { inter } (\mathcal {E})$ have two important properties: every interleaving in $\mathop { inter } (\mathcal {E})$ is a run of *P*, and any two interleavings $\sigma , \sigma ' \in \mathop { inter } (\mathcal {E})$ reach the same state $\mathop { state } (\sigma )= \mathop { state } (\sigma ')$.

**Fig. 3.**
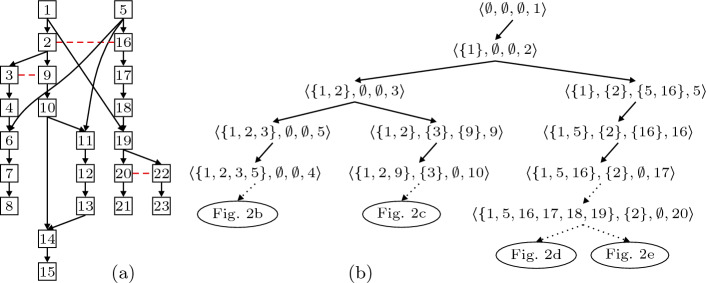
(a): unfolding of the program in Fig. 2a; (b): its POR exploration tree.

### Prime Event Structures

We use unfoldings to give semantics to multi-threaded programs. Unfoldings are Prime Event Structures [37], tree-like representations of system behavior that use partial orders to represent concurrent interaction.

Figure 3a depicts an unfolding of the program in Fig. 2a. The nodes are events and solid arrows represent causal dependencies: events 1 and 4 must fire before 8 can fire. The dotted line represents conflicts: 2 and 5 are not in conflict and may occur in any order, but 2 and 16 are in conflict and cannot occur in the same (partial-order) run.

Formally, a *Prime Event Structure* [37] (PES) is a tuple $\mathcal {E}\mathrel {:=}\langle E, {<}, {\mathrel {\#}}, h\rangle$ with a set of events *E*, a causality relation ${<} \subseteq E \times E$, which is a strict partial order, a conflict relation ${\mathrel {\#}} \subseteq E \times E$ that is symmetric and irreflexive, and a labeling function $h :E \rightarrow A$.

The *causes* of an event $\left\lceil e \right\rceil \mathrel {:=}{\{ e' \in E :e' < e \mathclose \}}$ are the least set of events that must fire before *e* can fire. A *configuration* of $\mathcal {E}$ is a finite set $C \subseteq E$ that is causally closed ($\left\lceil e \right\rceil \subseteq C$ for all $e \in C$), and conflict-free ($\lnot (e \mathrel {\#}e')$ for all $e, e' \in C$). We let $\mathop { conf } (\mathcal {E})$ denote the set of all configurations of $\mathcal {E}$. For any $e \in E$, the *local configuration* of *e* is defined as $[e] \mathrel {:=}\left\lceil e \right\rceil \cup {\{ e \mathclose \}}$. In Fig. 3a, the set ${\{ 1,2 \mathclose \}}$ is a configuration, and in fact it is a local configuration, i.e., $[2] = {\{ 1,2 \mathclose \}}$. The local configuration of event 6 is ${\{ 1,2,3,4,5,6 \mathclose \}}$. Set ${\{ 2,5,16 \mathclose \}}$ is not a configuration, because it is neither causally closed (1 is missing) nor conflict-free ($2 \mathrel {\#}16$).

### Unfolding Semantics for Programs

Given a program *P*, in this section we define a PES $\mathcal {U}_{P}$ such that every configuration of $\mathcal {U}_{P}$ is a partial-order run of *P*.

Let $\mathcal {E}_1 \mathrel {:=}\langle E_1, {<}_1, h_1\rangle , \ldots , \mathcal {E}_n \mathrel {:=}\langle E_n, {<}_n, h_n\rangle$ be the collection of all the partial-order runs of *P*. The events of $\mathcal {U}_{P}$ are the equivalence classes of the structural equality relation that we intuitively described in Sect. 2.3.

Two events are structurally equal iff their *canonical name* is the same. Given some event $e \in E_i$ in some partial-order run $\mathcal {E}_i$, the canonical name $\mathop { cn } (e)$ of *e* is the pair $\langle a, H\rangle$ where $a \mathrel {:=}h_i(e)$ is the executed action and $H \mathrel {:=}{\{ \mathop { cn } (e') :e' <_i e \mathclose \}}$ is the set of canonical names of those events that causally precede *e* in $\mathcal {E}_i$. Intuitively, canonical names indicate that action *h*(*e*) runs after the (transitively canonicalized) partially-ordered history preceding *e*. For instance, in Fig. 3a for events 1 and 6 we have $\mathop { cn } (1) = \langle \langle 1, \langle \textsf {loc}, {\texttt {a=in()}}\rangle \rangle , \emptyset \rangle$, and $\mathop { cn } (6) = \langle \langle 2, \langle \textsf {acq}, m\rangle \rangle , {\{ \mathop { cn } (1), \mathop { cn } (2), \mathop { cn } (3), \mathop { cn } (4), \mathop { cn } (5) \mathclose \}}\rangle$. Actually, the number within every event in Fig. 2b to 2e identifies (is in bijective correspondence with) its canonical name. Event 19 in Fig. 2d is the same event as event 19 in Fig. 2e because it fires the same action ($\langle 1, \langle \textsf {acq}, m\rangle \rangle$) after the same causal history (${\{ 1,5,16,17,18 \mathclose \}}$). Event 2 in Fig. 2c and 19 in Fig. 2d are *not* the same event because while $h(2) = h(19) = \langle 1, \langle \textsf {acq}, m\rangle \rangle$ they have a different causal history (${\{ 1 \mathclose \}}$ vs. ${\{ 1,5,16,17,18 \mathclose \}}$). Obviously events 4 and 6 in Fig. 2b are different because $h(4) \ne h(6)$. We can now define the *unfolding* of *P* as the only PES $\mathcal {U}_{P} \mathrel {:=}\langle E, {<}, \mathrel {\#}, h\rangle$ such that

- $E \mathrel {:=}{\{ \mathop { cn } (e) :e \in E_1 \cup \ldots \cup E_n \mathclose \}}$ is the set of canonical names of all events;
- Relation ${<} \subseteq E \times E$ is the union ${<_1} \cup \ldots \cup {<_n}$ of all happens-before relations;
- Any two events $e, e' \in E$ of $\mathcal {U}_{P}$ are in conflict, $e \mathrel {\#}e'$, when $e \ne e'$, and $\lnot (e < e')$, and $\lnot (e' < e)$, and *h*(*e*) is dependent on $h(e')$.

Figure 3a shows the unfolding produced by merging all 4 partial-order runs in Fig. 2b to 2e. Note that the configurations of $\mathcal {U}_{P}$ are partial-order runs of *P*. Furthermore, the $\subseteq$-maximal configurations are exactly the 4 originating partial orders. It is possible to prove that $\mathcal {U}_{P}$ is a semantics of *P*. In [42] we show that (1) $\mathcal {U}_{P}$ is uniquely defined, (2) any interleaving of any local configuration of $\mathcal {U}_{P}$ is a run of *P*, (3) for any run $\sigma$ of *P* there is a configuration *C* of $\mathcal {U}_{P}$ such that $\sigma \in \mathop { inter } (C)$.

### Conflicting Extensions

Our technique analyzes *P* by iteratively constructing (all) partial-order runs of *P*. In every iteration we need to find the next partial order to explore. We use the so-called *conflicting extensions* of a configuration to detect how to start a new partial-order run that has not been explored before.

Given a configuration *C* of $\mathcal {U}_{P}$, an *extension* of *C* is any event $e \in E \setminus C$ such that all the causal predecessors of *e* are in *C*. We denote the set of extensions of *C* as $\mathop { ex } (C) \mathrel {:=}{\{ e \in E :e \notin C \wedge \left\lceil e \right\rceil \subseteq C \mathclose \}}$. The *enabled* events of *C* are extensions that can form a larger configuration: $\mathop { en } (C) \mathrel {:=}{\{ e \in \mathop { ex } (C) :C \cup {\{ e \mathclose \}} \in \mathop { conf } (\mathcal {E}) \mathclose \}}$. For instance, in Fig. 3a, the (local) configuration [6] has 3 extensions, $\mathop { ex } ([6]) = {\{ 7,9,16 \mathclose \}}$ of which, however, only event 7 is enabled: $\mathop { en } ([6]) = {\{ 7 \mathclose \}}$. Event 19 is not an extension of [6] because 18 is a causal predecessor of 19, but $18 \not \in [6]$. A *conflicting extension* of *C* is an extension for which there is at least one $e' \in C$ such that $e \mathrel {\#}e'$. The (local) configuration [6] from our previous example has two conflicting extensions, events 9 and 16. A conflicting extension is, intuitively, an incompatible addition to the configuration *C*, an event *e* that cannot be executed together with *C* (without removing $e'$ and its causal successors from *C*). We denote by $\mathop { cex } (C)$ the set of all conflicting extensions of *C*, which coincides with the set of all extensions that are not enabled: $\mathop { cex } (C) \mathrel {:=}\mathop { ex } (C) \setminus \mathop { en } (C)$.

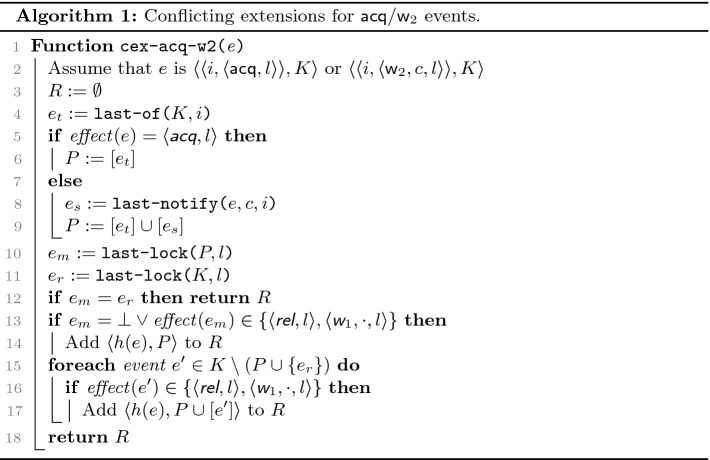



Our technique discovers new conflicting extension events by trying to revert the causal order of certain events in *C*. Owing to space limitations we only explain how the algorithm handles events of $\textsf {acq}$ and $\textsf {w}_2$ effect ( [42] presents the remaining 4 procedures of the algorithm). Algorithm 1 shows the procedure that handles this case. It receives an event *e* of $\textsf {acq}$ or $\textsf {w}_2$ effect (line 2). We build and return a set of conflicting extensions, stored in variable *R*. Events are added to *R* in line 14 and 17. Note that we define events using their canonical name. For instance, in line 14 we add a new event whose action is *h*(*e*) and whose causal history is *P*. Note that we only create events that execute action *h*(*e*). Conceptually speaking, the algorithm simply finds different causal histories (variables *P* and $e'$) within the set $K = \left\lceil e \right\rceil$ to execute action *h*(*e*).

Procedure last-of(*C*, *i*) returns the only <-maximal event of thread *i* in *C*; last-notify(*e*, *c*, *i*) returns the only immediate <-predecessor $e'$ of *e* such that the effect of $h(e')$ is either $\langle \textsf {sig},c,i\rangle$ or $\langle \textsf {bro},c,S\rangle$ with $i \in S$; finally, procedure last-lock(*C*, *l*) returns the only <-maximal event that manipulates lock *l* in *C* (an event of effect $\textsf {acq}$, $\textsf {rel}$, $\textsf {w}_1$ or $\textsf {w}_2$), or $\bot$ if no such event exists. See [42] for additional details.

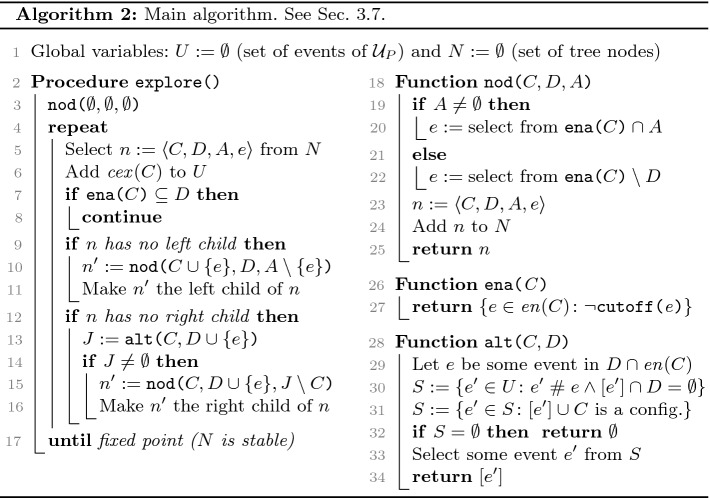



### Exploring the Unfolding

This section presents an algorithm that explores the state space of *P* by constructing all maximal configurations of $\mathcal {U}_{P}$. In essence, our procedure is an improved Quasi-Optimal POR algorithm [35], where the unfolding is not explored using a DFS traversal, but a user-defined search order. This enables us to build upon the preexisting exploration heuristics (“searchers”) in KLEE rather than having to follow a strict DFS exploration of the unfolding.

Our algorithm explores one configuration of $\mathcal {U}_{P}$ at a time and organizes the exploration into a binary tree. Figure 3b shows the tree explored for the unfolding shown in Fig. 3a. A tree node is a tuple $n \mathrel {:=}\langle C,D,A,e\rangle$ that represents both the exploration of a configuration *C* of $\mathcal {U}_{P}$ and a choice to execute, or not, event $e \in \mathop { en } (C)$. Both *D* (for *disabled*) and *A* (for *add*) are sets of events.

The key insight of this tree is as follows. The subtree rooted at a given node *n* explores all configurations of $\mathcal {U}_{P}$ that include *C* and exclude *D*, with the following constraint: *n*’s left subtree explores all configurations including event *e* and *n*’s right subtree explores all configuration excluding *e*. Set *A* is used to guide the algorithm when exploring the right subtree. For instance, in Fig. 3b the subtree rooted at node $n \mathrel {:=}\langle {\{ 1,2 \mathclose \}},\emptyset ,\emptyset ,3\rangle$ explores all maximal configurations that contain events 1 and 2 (namely, those shown in Fig. 2b and 2c). The left subtree of *n* explores all configurations including ${\{ 1,2,3 \mathclose \}}$ (Fig. 2b) and the right subtree all of those including ${\{ 1,2 \mathclose \}}$ but excluding 3 (Fig. 2c).

Algorithm 2 shows a simplified version of our algorithm. The complete version, in [42], specifies additional details including how nodes are selected for exploration and how they are removed from the tree. The algorithm constructs and stores the exploration tree in the variable $N$, and the set of currently known events of $\mathcal {U}_{N}$ in variable $U$. At the end of the exploration, $U$ will store all events of $\mathcal {U}_{N}$ and the leafs of the exploration tree in *N* will correspond to the maximal configurations of $\mathcal {U}_{N}$.

The tree is constructed using a fixed-point loop (line 4) that repeats the following steps as long as they modify the tree: select a node $\langle C,D,A,e\rangle$ in the tree (line 5), extend *U* with the conflicting extensions of *C* (line 6), check if the configuration is $\subseteq$-maximal (line 7), in which case there is nothing left to do, then try to add a left (line 9) or right (line 12) child node.

The subtree rooted at the left child node will explore all configurations that include $C \cup {\{ e \mathclose \}}$ and exclude *D* (line 10); the right subtree will explore those including *C* and excluding $D \cup {\{ e \mathclose \}}$ (line 15), if any of them exists, which we detect by checking (line 14) if we found a so-called *alternative* [41].

An alternative is a set of events which witnesses the existence of some maximal configuration in $\mathcal {U}_{P}$ that extends *C* without including $D \cup {\{ e \mathclose \}}$. Computing such witness is an NP-complete problem, so we use an approximation called *k-partial alternatives* [35], which can be computed in P-time and works well in practice. Our procedure alt specifically computes 1-partial alternatives: it selects $k=1$ event *e* from $D \cap \mathop { en } (C)$, searches for an event $e'$ in conflict with *e* (we have added all known candidates in line 6, using the algorithms of Sect. 3.6) that can extend *C* (i.e., such that $C \cup [e']$ is a configuration), and returns it. When such an event $e'$ is found (line 33), some events in its local configuration $[e']$ become the *A*-component of the right child node (line 15), and the leftmost branch rooted at that node will re-execute those events (as they will be selected in line 20), guiding the search towards the witnessed maximal configuration.

For instance, in Fig. 3b, assume that the algorithm has selected node $n = \langle {\{ 1 \mathclose \}}, \emptyset , \emptyset , 2\rangle$ at line 5 when event 16 is already in *U*. Then a call to alt( ${\{ 1 \mathclose \}}, {\{ 2 \mathclose \}}$) is issued at line 13, event $e = 2$ is selected at line 29 and event $e' = 16$ gets selected at line 33, because $2 \mathrel {\#}16$ and $[16] \cup {\{ 1 \mathclose \}}$ is a configuration. As a result, node $n' = \langle {\{ 1 \mathclose \}}, {\{ 2 \mathclose \}}, {\{ 5,16 \mathclose \}}, 5\rangle$ becomes the right child of *n* in line 15, and the leftmost branch rooted at $n'$ adds ${\{ 5,16 \mathclose \}}$ to *C*, leading to the maximal configuration Fig. 2d.

### Cutoffs and Completeness

All interleavings of a given configuration always reach the same state, but interleavings of different configurations can also reach the same state. It is possible to exclude certain such redundant configurations from the exploration without making the algorithm incomplete, by using *cutoff* events [32].

Intuitively, an event is a cutoff if we have already visited another event that reaches the same state with a shorter execution. Formally, in Algorithm 2, line 27 we let cutoff(*e*) return *true* iff there is some $e' \in U$ such that $\mathop { state } ([e]) = \mathop { state } ([e'])$ and $|[e']| < |[e]|$. This makes Algorithm 2 ignore cutoff events and any event that causally succeeds them. Sect. 4.2 explains how to effectively implement the check $\mathop { state } ([e]) = \mathop { state } ([e'])$.

While cutoffs prevent the exploration of redundant configurations, the analysis is still complete: it is possible to prove that every state reachable via a configuration with cutoffs is also reachable via a configuration without cutoffs. Furthermore, cutoff events not only reduce the exploration of redundant configurations, but also force the algorithm to terminate for non-terminating programs that run on bounded memory.

#### Theorem 2 (Correctness)

For any reachable state $s \in \mathop { reach } (P)$, Algorithm 2 explores a configuration *C* such that for some $C' \subseteq C$ it holds that $\mathop { state } (C') = s$. Furthermore, it terminates for any program *P* such that $\mathop { reach } (P)$ is finite.

A proof sketch is available in [42]. Naturally, since Algorithm 2 explores $\mathcal {U}_{P}$, and $\mathcal {U}_{P}$ is an exact representation of all runs of *P*, then Algorithm 2 is also *sound*: any event constructed by the algorithm (added to set *U*) is associated with a real run of *P*.

## Implementation

We implemented our approach on top of the symbolic execution engine KLEE [10], which was previously restricted to sequential programs. KLEE already provides a minimal POSIX support library that we extended to translate calls to pthread functions to their respective actions, enabling us to test real-world multi-threaded C programs. We also extended already available functionality to make it thread-safe, e.g., by implementing a global file system lock that ensures that concurrent reads from the same file descriptor do not result in unsafe behavior. The source code of our prototype is available at https://github.com/por-se/por-se.

### Standby States

When a new alternative is explored, a symbolic execution state needs to be computed to match the new node in the POR tree. However, creating it from scratch requires too much time and keeping a symbolic execution state around for each node consumes significant amounts of memory. Instead of committing to either extreme, we store *standby states* at regular intervals along the exploration tree and, when necessary, replay the closest standby state. This way, significantly fewer states are kept in memory without letting the replaying of previously computed operations dominate the analysis either.

### Hash-Based Cutoff Events

Schemmel et al. presented [43] an incremental hashing scheme to identify infinite loops during symbolic execution. The approach detects when the program under test can transition from any one state back to that same state. Their scheme computes *fragments* for small portions of the program state, which are then hashed individually, and combined into a compound hash by bitwise xor operations. This compound hash, called a *fingerprint*, uniquely (modulo hash collisions) identifies the whole state of the program under test. We adapt this scheme to provide hashes that identify the concurrent state of parallel programs.

To this end, we associate each configuration with a fingerprint that describes the whole state of the program at that point. For example, if the program state consists of two variables, $x=3$ and $y=5$, the fingerprint would be $fp=\text {hash}\left( {\texttt {"x=3"}}\right) \oplus \text {hash}\left( {\texttt {"y=5"}}\right)$. When one fragment changes, e.g., from $x=3$ to $x=4$, the old fragment hash needs to be replaced with the new one. This operation can be performed as $fp'=fp\oplus \text {hash}\left( {\texttt {"x=3"}}\right) \oplus \text {hash}\left( {\texttt {"x=4"}}\right)$ as the duplicate fragments for $x=3$ will cancel out. To quickly compute the fingerprint of a configuration, we annotate each event with an xor of all of these update operations that were done on its thread. Computing the fingerprint of a configuration now only requires xor-ing the values from its thread-maximal events, which will ensure that all changes done to each variable are accounted for, and cancel out one another so that only the fragment for the last value remains.

Any two local configurations that have the same fingerprint represent the same program state; each variable, program counter, etc., has the same value. Thus, it is not necessary to continue exploring both—we have found a potential cutoff point, which the POR algorithm will treat accordingly (Sect. 3.8).

### Deterministic and Repeatable Allocations

KLEE usually uses the system allocator to determine the addresses of objects allocated by the program under test. But it also provides a (more) deterministic mode, in which addresses are consumed in sequence from a large pre-allocated array. Since our hash-based cutoff computation uses memory address as part of the computation, using execution replays from standby states (Sect. 4.1) requires that we have fully repeatable memory allocation.

We tackle this problem by decoupling the addresses returned by the emulated system allocator in the program under test from the system allocator of KLEE itself. A new allocator requires a large amount of virtual memory in which it will perform its allocations. This large virtual memory mapping is not actually used unless an external function call is performed, in which case the relevant objects are temporarily copied into the region from the symbolic execution state for which the external function call is to be performed. Afterwards, the pages are marked for reclamation by the OS. This way, allocations done by different symbolic execution states return the same address to the program under test.

While a deterministic allocator by itself would be enough for providing deterministic allocation to sequential programs, parallel programs also require an allocation pattern that is independent of which sequentialization of the same partial order is chosen. We achieve this property by providing independent allocators for each thread (based on the thread id, thus ensuring that the same virtual memory mapping is reused for each instance of the same semantic thread). When an object is deallocated on a different thread than it was allocated on, its address only becomes available for reuse once the allocating thread has reached a point in its execution where it is causally dependent on the deallocation. Additionally, the thread ids that are used by our implementation are hierarchically defined: A new thread $t$ that is the $i$-th thread started by its parent thread $p$ has the thread id $t := \left( p,i\right)$, with the main thread being denoted as $\left( 1\right)$. This way, thread ids and the associated virtual memory mappings are independent of how the concurrent creation of multiple threads are sequentialized.

We have also included various optimizations that promote controlled reuse of addresses to increase the chance that a cutoff event (Sect. 4.2) is found, such as binning allocations by size, which reduces the chance that temporary allocations impact which addresses are returned for other allocations.

### Data Race Detection

Our data race detection algorithm simply follows the happens-before relationships established by the POR. However, its implementation is complicated by the possibility of addresses becoming symbolic. Generally speaking, a symbolic address can potentially point to any and every byte in the whole address space, thus requiring frequent and large SMT queries to be solved.

To alleviate the quadratic blowup of possibly aliasing accesses, we exploit how KLEE performs memory accesses with symbolic addresses: The symbolic state is forked for every possible memory object that the access may refer to (and one additional time if the memory access may point to unallocated memory). Therefore, a symbolic memory access is already resolved to memory object granularity when it potentially participates in a data race. This drastically reduces the amount of possible data races without querying the SMT solver.

### External Function Calls

When a program wants to call a function that is neither provided by the program itself nor by the runtime, KLEE will attempt to perform an *external function call* by moving the function arguments from the symbolic state to its own address space and attempting to call the function itself. While this support for uninterpreted functions is helpful for getting some results for programs which are not fully supported by KLEE’s POSIX runtime, it is also inherently incomplete and not sound in the general case. Our prototype includes this option as well.

## Experimental Evaluation

To explore the efficacy of the presented approach, we performed a series of experiments including both synthetic benchmarks from the SV-COMP [9] benchmark suite and real-world programs, namely, Memcached [3] and GNU sort [1]. We compare against Yogar-CBMC [49], which is the winner of the concurrency safety category of SV-COMP 2019 [9], and stands in for the family of bounded model checkers. As such, Yogar-CBMC is predestined to fare well in the artificial SV-COMP benchmarks, while our approach may demonstrate its strength in dealing with more complicated programs.

**Table 1.** Our prototype and Yogar-CBMC running SV-COMP benchmarks. Timeout set at 15 min with maximum memory usage of 15 GB. Columns are: T: true result, output matches expected verdict; F: false result, output does not match expected verdict; U: unknown result, tool yields no answer; Time: total time taken; RSS: maximum resident set size over all benchmarks.

Benchmark	Our tool					Yogar-CBMC				
	T	F	U	Time	RSS	T	F	U	Time	RSS
pthread	29	–	9	1:50:19	16 GB	29	–	9	0:31:21	948 MB
pthread-driver-races	16	1	4	1:03:08	6049 MB	21	–	–	0:00:12	72 MB

We ran the experiments on a cluster of multiple identical machines with dual Intel Xeon E5-2643 v4 CPUs and 256 GiB of RAM. We used a 4 h timeout and 200 GB maximum memory usage for real-world programs. We used a 15 min timeout and 15 GB maximum memory for individual SV-COMP benchmarks.

### SV-COMP

We ran our tool and Yogar-CBMC on the “pthread” and “pthread-driver-races” benchmark suites in their newest (2020) incarnation. As expected, Table 1 shows that Yogar-CBMC clearly outperforms our tool for this specific set of benchmarks. Not only does Yogar-CBMC not miscategorize even a single benchmark, it does so quickly and without using a lot of memory. Our tool, in contrast, takes significantly more time and memory to analyze the target benchmarks. In fact, several benchmarks do not complete within the 15 min time frame and therefore cannot give a verdict for those.

The “pthread-driver-races” benchmark suite contains one benchmark that is marked as a failure for our tool in Table 1. For the relevant benchmark, a verdict of “target function unreachable” is expected, which we translate to mean “no data race occurs”. However, the benchmark program constructs a pointer that may point to effectively any byte in memory, which, upon dereferencing it, leads to both, memory errors and data races (by virtue of the pointer also being able to touch another thread’s stack). While we report this behavior for completeness sake, we attribute it to the adaptations made to fit the SV-COMP model to ours.

**Preparation of Benchmark Suites.** The SV-COMP benchmark suite does not only assume various kinds of special casing (e.g., functions whose name begins with  must be executed atomically), but also routinely violates the C standard by, for example, employing data races as a control flow mechanism [25, § 5.1.2.4/35]. Partially, this is because the analysis target is a question of reachability of a certain part of the benchmark program, not its correctness. We therefore attempted to guess the intention of the individual benchmarks, making variables atomic or leaving the data race in when it is the aim of the benchmark.

### Memcached

Memcached [3] is an in-memory network object cache written in C. As it is a somewhat large project with a fairly significant state space, we were unable to analyze it completely, even though our prototype still found several bugs. Our attempts to run Yogar-CBMC did not succeed, as it reproducibly crashes.

**Faults Detected.** Our prototype found nine bugs in memcached 1.5.19, attributable to four different root causes, all of which where previously unknown. The first bug is a misuse of the pthread API, causing six mutexes and condition variables to be initialized twice, leading to undefined behavior. We reported^2^ the issue, a fix is included in version 1.5.20. The second bug occurs during the initialization of memcached, where fields that will later be accessed in a thread-safe manner are sometimes accessed in a non-thread-safe manner, assuming that competing accesses are not yet possible. We reported^3^ a mistake our tool found in the initialization order that invalidates the assumption that locking is not (yet) necessary on one field. A fix ships with memcached 1.5.21. For the third bug, memcached utilizes a maintenance thread to manage and resize its core hash table when necessary. Additionally, on another thread, a timer checks whether the maintenance thread should perform an expansion of the hash table. We found^4^ a data race between these two threads on a field that stores whether the maintenance thread has started expanding. This is fixed in version 1.5.20. The fourth and final issue is a data race on the stats_state storing execution statistics. We reported^5^ this issue and a fix is included in version 1.5.21.

**Experiment.** We run our prototype on five different versions of memcached, the three releases 1.5.19, 1.5.20 and 1.5.21 plus variants of the earlier releases (1.5.19+ and 1.5.20+) which include patches for the two bugs we found during program initialization. Those variants are included to show performance when not restricted by inescapable errors very early in the program execution.

Table 2 shows clearly how the two initialization bugs may lead to very quick analyses—versions 1.5.19 and 1.5.20 are completely analyzed in 7 s each, while versions 1.5.19+, 1.5.20+ and 1.5.21 exhaust the memory budget of 200 GB. We have configured the experiment to stop the analysis once the memory limit is reached, although the analysis could continue in an incomplete manner by removing parts of the exploration frontier to free up memory. Even though the number of error paths in Table 2 differs between configurations, it is notable that each configuration can only reach exactly one of the bugs, as execution is arrested at that point. When not restricted to the program initialization, the analysis of memcached produces hundreds of thousands of events and retires hundreds of millions of instructions in less than 2 h.

**Table 2.** Our prototype analyzing various versions of memcached and GNU sort. Timeout set at 4 h with maximum memory usage of 200 GB. Columns are: RSS: maximum resident set size (swap space is not available); #I: number of instructions executed; Th: maximum number of threads active at the same time;  : total number of events in the explored unfolding; Mut: number of mutex lock/unlock events; CV: number of wait1/wait2/signal/broadcast events;  : number of symbolic choices; Cut: number of events determined to be cutoffs; and the number of Finished Paths distinguish between normal termination of the program under test (Exit), detection of an error (Err) and being cut off (Cut).

Program			Performance			Th	Events					Finished Paths			Halt
	Version	LoC	Time	RSS	#I			Mut	CV		Cut	Exit	Err	Cut	Reason
Memcached	1.5.19	31065	0:00:07	204 MB	23K	1	12	6	0	3	0	0	1	0	Finished
	1.5.19+	31051	1:33:42	208 GB	1.2B	6	331K	271K	60K	3	24K	0	41K	29K	Memory
	1.5.20	31093	0:00:07	197 MB	92K	2	24	16	0	3	0	0	1	0	Finished
	1.5.20+	31093	1:51:10	207 GB	228M	10	745K	742K	2.7K	5	882	0	1	2.6K	Memory
	1.5.21	31090	1:29:57	207 GB	546M	10	1.1M	1.1M	3.1K	3	558	0	0	2.6K	Memory
Sort	8.31	86596	0:24:29	23 GB	266M	2	1.8M	1.4M	269K	25K	58K	8.0K	4.9K	55K	Finished
	8.31+	86599	4:01:39	88 GB	1.0B	2	6.9M	5.8M	777K	276K	346K	6.3K	0	285K	Time

Our setup delivers a single symbolic packet to memcached followed by a concrete shutdown packet. As this packet can obviously only be processed once the server is ready to process input, we observe symbolic choices only after program startup is complete. (Since our prototype builds on KLEE, note that it assumes a single symbolic choice during startup, without generating an additional path.)

### GNU sort

GNU sort uses threads for speeding up the sorting of very large workloads. We reduced the minimum size of input required to trigger concurrent sorting to four lines to enable the analysis tools to actually trigger concurrent behavior. Nevertheless, we were unable to avoid crashing Yogar-CBMC on this input.

During analysis of GNU sort 8.31, our prototype detected a data race, that we manually verified, but were unable to trigger in a harmful manner. Table 2 shows two variants of GNU sort, the baseline version with eager parallelization (8.31) and a version with added locking to prevent the data race (8.31+).

Surprisingly, version 8.31 finishes the exploration, as all paths either exit, encounter the data race and are terminated or are cut off. By fixing the data race in version 8.31+, we make it possible for the exploration to continue beyond this point, which results in a full 4 h run that retires a full billion instructions while encountering almost seven million unique events.

## Related Work

The body of work in *systematic concurrency testing* [5, 6, 19, 21, 23, 35, 41, 47, 50] is large. These approaches explore thread interleavings under a fixed program input. They prune the search space using context-bounding [34], increasingly sophisticated PORs [5–7, 12, 19, 23, 35, 41], or random testing [13, 50]. Our main difference with these techniques is that we handle input data.

Thread-modular abstract interpretation [18, 30, 33] and unfolding-based abstract interpretation [46] aim at proving safety rather than finding bugs. They use over-approximations to explore all behaviors, while we focus on testing and never produce false alarms. *Sequentialization* techniques [26, 36, 40] encode a multi-threaded program into a sequential one. While these encodings can be very effective for small programs [26] they grow quickly with large context bounds (5 or more, see [36]). However, some of the bugs found by our technique (Sect. 5) require many context switches to be reached.

*Bounded-model checking* [8, 15, 28, 39, 49] for multi-threaded programs encode multiple program paths into a single logic formula, while our technique encodes a single path. Their main disadvantage is that for very large programs, even constructing the multi-path formula can be extremely challenging, often producing an upfront failure and no result. Conversely, while our approach faces path explosion, it is always able to test some program paths.

Techniques like [17, 27, 44] operate on a data structure conceptually very similar to our unfolding. They track read/write operations to every variable, which becomes a liability on very large executions. In contrast, we only use POSIX synchronization primitives and compactly represent memory accesses to detect data races. Furthermore, they do not exploit anything similar to cutoff events for additional trace pruning.

Interpolation [14, 48] and weakest preconditions [24] have been combined with POR and symbolic execution for *property-guided* analysis. These approaches are mostly complementary to PORs like our technique, as they eliminate a different class of redundant executions [24].

This work builds on top of previous work [35, 41, 46]. The main contributions w.r.t. those are: (1) we use symbolic execution instead of concurrency testing [35, 41] or abstract interpretation [46]; (2) we support condition variables, providing algorithms to compute conflicting extensions for them; and (3) here we use hash-based fingerprints to compute cutoff events, thus handling much more complex partial orders than the approach described in [46].

## Conclusion

Our approach combines POR and symbolic execution to analyze programs w.r.t. both input (data) and concurrency non-determinism. We model a significant portion of the pthread API, including try-lock operations and robust mutexes. We introduce two techniques to cope with state-space explosion in real-world programs. We compute cutoff events by using efficiently-computed fingerprints that uniquely identify the total state of the program. We restrict scheduling to synchronization points and report data races as errors. Our experiments found previously unknown bugs in real-world software projects (memcached, GNU sort).

## Footnotes

1 The formal definition is slightly more complex, see [42] for the details.

2 https://github.com/memcached/memcached/pull/566.

3 https://github.com/memcached/memcached/pull/575.

4 https://github.com/memcached/memcached/pull/569.

5 https://github.com/memcached/memcached/pull/573.
